# Managing diagnostic uncertainty in primary care: a systematic critical review

**DOI:** 10.1186/s12875-017-0650-0

**Published:** 2017-08-07

**Authors:** Rahul Alam, Sudeh Cheraghi-Sohi, Maria Panagioti, Aneez Esmail, Stephen Campbell, Efharis Panagopoulou

**Affiliations:** 1 NIHR Greater Manchester Primary Care Patient Safety Translational Research Centre (Greater Manchester PSTRC), The University of Manchester, Williamson Building, Oxford Road, Manchester, M13 9PL UK; 2 Centre for Research and Action in Public Health, University of Canberra, ACT, 2601 Australia; 3 Medical School, Department Social Medicine, Aristotle University, Thessaloniki, Greece

**Keywords:** Diagnosis, Uncertainty, Primary medical care, Burnout, Training

## Abstract

**Background:**

Diagnostic uncertainty is one of the largest contributory factors to the occurrence of diagnostic errors across most specialties in medicine and arguably uncertainty is greatest in primary care due to the undifferentiated symptoms primary care physicians are often presented with. Physicians can respond to diagnostic uncertainty in various ways through the interplay of a series of cognitive, emotional and ethical reactions. The consequences of such uncertainty however can impact negatively upon the primary care practitioner, their patients and the wider healthcare system. Understanding the nature of the existing empirical literature in relation to managing diagnostic uncertainty in primary medical care is a logical and necessary first step in order to understand what solutions are already available and/or to aid the development of any training or feedback aimed at better managing this uncertainty. This review is the first to characterize the existing empirical literature on managing diagnostic uncertainty in primary care.

**Methods:**

Sixteen databases were systematically searched from inception to present with no restrictions. Hand searches of relevant websites and reference lists of included studies were also conducted. Two authors conducted abstract/article screening and data extraction. PRISMA guidelines were adhered to.

**Results:**

Ten studies met the inclusion criteria. A narrative and conceptual synthesis was undertaken under the premises of critical reviews. Results suggest that studies have focused on internal factors (traits, skills and strategies) associated with managing diagnostic uncertainty with only one external intervention identified. Cognitive factors ranged from the influences of epistemological viewpoints to practical approaches such as greater knowledge of the patient, utilizing resources to hand and using appropriate safety netting techniques. Emotional aspects of uncertainty management included clinicians embracing uncertainty and working with provisional diagnoses. Ethical aspects of uncertainty management centered on communicating diagnostic uncertainties with patients. Personality traits and characteristics influenced each of the three domains.

**Conclusions:**

There is little empirical evidence on how uncertainty is managed in general practice. However we highlight how the extant literature can be conceptualised into cognitive, emotional and ethical aspects of uncertainty which may help clinicians be more aware of their own biases as well as provide a platform for future research.

**Trial registration:**

PROSPERO registration: CRD42015027555

**Electronic supplementary material:**

The online version of this article (doi:10.1186/s12875-017-0650-0) contains supplementary material, which is available to authorized users.

## Background

Diagnostic uncertainty is one of the largest contributory factors to the occurrence of diagnostic errors across most specialties in medicine [1]. It is also the most common source of clinical uncertainty in primary care, as the breadth and complexity of diagnoses possible in general practice makes diagnostic uncertainty a routine inevitability [2–4]. Although diagnostic uncertainty is primarily a function of knowledge acquisition, processing and recall [4], physicians can respond to diagnostic uncertainty in various ways through the interplay of a series of cognitive, emotional and ethical reactions [5–7].

Cognitive reactions are related to difficulties in perception and interpretation of the available facts [8] and often include the use of several heuristics and biases such as unconscious “rules of thumb” or intuitive diagnostic reasoning based on stereotypes related to gender, age or occupation [9, 10].

Emotional reactions include both short and long-term stress and/or anxiety which can develop under particular uncertain or ambiguous situations and contexts [11]. They include the cultural and/or societal context in which clinical uncertainty take place (where certainty is expected and demanded by patients) alongside the affective reactions clinicians experience when their technical, personal, or conceptual resources are unable to meet the demand for certainty [4].

Ethical reactions relate to the nature of discourse between the physician and patient in relation to uncertainty and encompass elements of shared decision-making [12, 13]. Informed and shared decision-making necessitates patient understanding of their illness, options for treatment and prognosis [14]. Despite this, physicians are often hesitant to disclose uncertainty to their patients [15] leaving clinicians in an ethical dilemma.

At a dyadic level, a physician’s ability to deal with uncertainty at a cognitive, emotional and ethical level influences the eventual diagnostic decision and therefore can impact on the patient and their outcomes [4]. At a wider system level, uncertainty has been shown to effect admission rates [16] and health care costs [17]. A recent study for example, has shown that General Practitioners (GPs) or family physicians respond to uncertainty by increased hospital referrals and ordering more diagnostic tests [18]. Doctors with a high intolerance of uncertainty also have higher costs of investigation and treatment [19]. Conversely, GPs who cope well with uncertainty are more likely to support shared decision-making [20]. Finally, by attempting to achieve certainty via a ‘correct diagnosis’, premature closure is likely to occur in the decision-making process thereby allowing hidden assumptions and unconscious biases to have more weight than they should, with increased potential for diagnostic error [21].

In summary, diagnostic uncertainty has implications for the primary care practitioner, their patients and the wider healthcare system. Understanding the nature of the existing empirical literature in relation to managing diagnostic uncertainty in primary medical care would be a logical and necessary first step in order to understand what solutions are already available and/or to aid the development of any training or feedback aimed at better managing this uncertainty.

The purpose of this systematic critical review therefore was to synthesize the strategies, skills or traits associated with or used by clinicians working in general medical practice to manage diagnostic uncertainty at any or all of the three domains of uncertainty (cognitive, emotional and/or ethical). Additionally, the review aimed to identify any existing training programs that aim to support clinicians to manage diagnostic uncertainty in order to inform the development of any future programs.

## Methods

This systematic critical review was conducted and reported in accordance with the Preferred Reporting Items for Systematic Reviews and Meta-Analyses (PRISMA) [22] and registered with PROSPERO in October 2015 (available online at: http://www.crd.york.ac.uk/PROSPERO/display_record.asp?ID=CRD42015027555).

### Eligibility criteria

Given that our focus was on primary medical care, any empirical studies reporting on how trainee or experienced GPs deal and manage with diagnostic uncertainty either wholly or partly in primary medical care were eligible. We also included studies where diagnostic uncertainty was included within broader clinical uncertainty assessments. Eligible study designs included randomised controlled trials, interrupted time series analysis, cohort studies, case control studies, cross-sectional studies, before and after studies, qualitative studies as well as pragmatic observational studies and studies such as process evaluations.

### Exclusion criteria

Studies involving solely secondary care contexts and student participants were excluded given the potentially limited generalizability to qualified clinicians in primary care as were studies exploring patient uncertainty. Programs on uncertainty that utilized alternative forms of minimizing diagnostic uncertainty such as the use of laboratory tests [23], utilising differential diagnosis (DDx) tools [24] and studies utilizing top-down approaches such as the use of guidelines on aspects of diagnostic uncertainty were also excluded.

### Information sources and searches

A combination of medical subject heading (MeSH) terms and free text words describing clinical uncertainty and tolerance were used. The following databases were searched from inception to July 2015: Ovid MEDLINE(R), Embase, CINAHL, Cochrane Database of Systematic Reviews, Cochrane Central Register of Controlled Trials, Cochrane Methodology Register, ACP Journal Club, Database of Abstracts of Reviews of Effects, Health Technology Assessment, National Health Service (NHS) Economic Evaluation Database, AMED (Allied and Complementary Medicine), CAB Abstracts, Global Health, Health and Psychosocial Instruments, Health Management Information Consortium and PsycINFO (see Additional file 1).

In addition to the articles identified by the search, we undertook hand-searches of the websites of the National Patient Safety Agency (NPSA), Patient.co.uk and Gutfeelings.eu. The reference lists of included articles were also screened for eligible papers and a Scopus search of potentially relevant articles were conducted using key words. The search was not restricted by date, language or country of publication.

### Study selection

Two reviewers (RA and EP) independently screened the titles and abstracts against the eligibility criteria for inclusion. The reviewers then agreed on the papers for inclusion and full-text articles were retrieved and reviewed. Any disagreements were resolved in meetings and through discussion with the wider team until consensus was reached. High inter-rater reliability was achieved: Cohen’s [25] unweighted κ coefficient = 0.74 and 0.95 for title/abstract and full-text screening, respectively.

### Data extraction

A standardized data extraction form was developed and piloted. Studies meeting the inclusion criteria were then double-extracted by the review team. Data were extracted and cross-checked by the reviewers using the Microsoft Excel data extraction form and any disagreements were resolved by discussion.

Data were extracted on the participants including their grade, clinical background, specialty and setting. We then extracted data on the specific types of diagnostic uncertainty targeted by the study (cognitive, emotional, or ethical), the tools/frameworks used to assess uncertainty and any resources utilized to manage diagnostic uncertainty (internal: i.e. skills, strategies, or individual traits, or external: i.e. interventions or training programs).

### Critical appraisal

The Critical Appraisal Skills Programme (CASP) checklist for qualitative research was used to assess the quality of the included qualitative studies [26]. The quantitative cross-sectional studies were critically appraised using the modified Newcastle Ottawa scale for cross sectional studies [27]. These two instruments are very well-known, easily accessible online and clearly define the meaning behind each individual criterion listed. We did not exclude studies from the synthesis based on the critical appraisal ratings.

### Data synthesis

As the main focus of this review was to explore which types of skills or strategies physicians use to manage diagnostic uncertainty under its three domains, a narrative synthesis was conducted drawing on the critical review synthesis methodology described by Grant and colleagues [28]. The main advantage of the critical review methodology is that it allows a conceptual synthesis to be undertaken which is particularly suited for the purposes of this study. Following the guidelines of a critical review, the studies were conceptually grouped into the cognitive, emotional and ethical domains of uncertainty which are widely described in the empirical and theoretical literature of uncertainty as outlined in the introduction. Within the main presentation groupings (the three domains of uncertainty), we also presented the findings according to the type of evidence (qualitative or quantitative data). The small number and the moderate quality of the included studies suggest that further groupings of the studies were very unlikely to make any firm contribution, and therefore were avoided.

## Results

### Overview

**Fig. 1:**
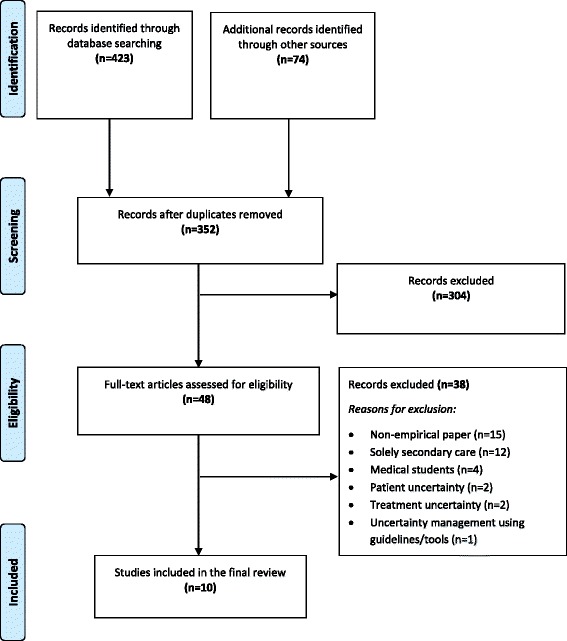
PRISMA flow diagram

We identified 10 studies meeting the inclusion criteria and the PRISMA flowchart demonstrates the study screening and selection process (please see Fig. 1).

### Descriptive characteristics of included studies and critical appraisal

Tables 1 and 2 show the characteristics of the included studies highlighting the traits, strategies and skills influencing and impacting on managing uncertainty. Six studies were quantitative cross-sectional surveys (Table 1) [4, 6, 10, 29–31], three were qualitative process evaluations [32–34] and one was a qualitative study [13] (Table 2). All three domains of diagnostic uncertainty were equally represented in the identified literature, with eight studies per domain. Some studies were categorized across more than one domain.

**Table 1:** Traits, strategies and skills influencing and impacting on managing uncertainty: Quantitative cross-sectional studies

Author Year Country	Study type	Specialty or condition/clinician grade or experience	Setting/ recruitment/ Sample size (n) and response rate (RR %)	Uncertainty assessment	Uncertainty Resource	Uncertainty type Cognitive (C) Emotional (E) Ethical (ETH)	Results
Cooke 2013 Australia [30]	Cross-sectional survey	General practice / Registrar	Survey participants recruited from advertisements and through training events; (*n* = 128) (RR: 90%)	Intolerance of uncertainty scale −12 (IUS-12) Physicians reactions to uncertainty Scale −8 (PRUS)	Resilience	General intolerance of uncertainty Anxiety due to uncertainty (E) Reluctance to disclose uncertainty to patients (Eth)	Lower resilience was associated with lower tolerance of uncertainty.
Evans 2009 USA [4]	Cross sectional survey	Pediatrics, family medicine and internal medicine/ Board certified and resident physicians in primary care with a mixture of experience levels	Primary care / Survey participants recruited from an academic medical centre (*n* = 78) (RR: 76%)	Physicians Belief Scale −32 (PBS), Physician reactions to uncertainty Scale – 8 (PRUS)	Cognitive beliefs	Conceptual uncertainty – difficulties applying abstract criteria to concrete situations (C) Stress reactions to uncertainty (E)	Physicians adopting a biopsychosocial epistemology model were associated with less stress reactions to uncertainty while a biomedical model was associated with more stress reactions to uncertainty. Clinician gender, specialty area and experience were correlated with stress reactions to uncertainty.
Nevalainen 2014 Finland [29]	Questionnaire-based survey (self-assessment)	Primary Care/Recently qualified GPs (<5 years, mean age 31.2 years) and experienced GPs (>5 years, mean age 48.4 years)	Convenience sample of GPs recruited via email. *N* = 165/244 (RR: 68%)	Custom made questionnaire	Experience	Access to information sources (C) Tolerance of uncertainty (E)	Experienced GPs better tolerate uncertainty (53.8% (95% CI; 42.2–65.0) in medical decision-making than their younger colleagues (25.9% (95% CI; 17.0–36.5) (*p* < 0.001).
Portnoy, 2011 USA [6]	Cross-sectional survey-representative sample	Mixed –Family practice, internists, pediatrics, obstetrics and gynecology / Mixed 13.9 years practicing (SD ± 7.5)	Primary care; *n* = 1500 total; (GPs *n* = 515 34.3%) (RR: 48%)	Ambiguity aversion (AA) in medicine scale.	Factors influencing physicians’ attitudes towards the communication and management of scientific uncertainty in clinical practice	Physicians’ attitudes about communicating and managing uncertainty and their perceptions of negative reactions to uncertainty by their patients (Eth)	Physician demographics (including medical specialty, ethnicity and gender) predicted attitudes towards communicating and managing scientific uncertainty. Physicians’ perceptions of their patients’ responses to ambiguity also influenced decisions to share ambiguity where physicians who thought more of their patients would have negative reactions to ambiguous information were more likely to decide what’s best for the patient (*p* = 0.013) and to withhold an intervention that had uncertainty associated with it (p = <0.001).
Schneider 2010 Germany [10]	Survey – questionnaire (development consisting of focus groups)	General Practice / Overall experience as a doctor –mean years =22.7, experience as a GP, 15.4 years	University hospital conference & 23 “quality circles” (GP groups). Responders *n* = 93 (conference) & *n* = 232 (quality circles) (Overall RR: 68%)	Developing the Dealing with uncertainty questionnaire (DUQ); GP diagnostic action scale and GP diagnostic reasoning scale.	Cognitive heuristics	Dealing with diagnostic uncertainty and heuristics in diagnostic thinking (C) Tolerance of uncertainty (E)	The use of test of time, knowledge of family situation and occupational situation as a simple heuristic. Emotional intolerance against uncertainty correlates with an increase in diagnostic activity. Emotional responses to uncertainty might influence gender-specific reactions to uncertainty in different ways.
Schneider 2014 Germany [31]	Mixed methods study (focus groups and cross sectional survey)	Primary care, generic conditions/ clinical experience 23.9 years (SD ± 23.9 years)	Development - 10 experienced GPs and survey - *n* = 228 (RR: 97%)	Communicating and dealing with uncertainty questionnaire (CoDU), Physician reaction to Uncertainty (PRU), and Big Five Inventory (BFI-K)	Personality traits of GPs in relation to decision making concerning uncertainty management	Diagnostic action (C) Intuition (E) Extended social anamnesis (Eth) Anxiety due to uncertainty (E) Communicating uncertainty (Eth)	GPs scoring high in neuroticism demonstrated more anxiety due to uncertainty and higher reluctance to communicate with patients. Extraversion, conscientiousness and openness correlated negatively with anxiety due to uncertainty and positively with patient communication.

*RR* Response rate, *SP* Standardized patients *NA* not applicable, *NS* not stated, *C* Cognitive, *E* emotional, *Eth* ethical

**Table 2:** Traits, strategies and skills influencing and impacting on managing uncertainty: Qualitative studies

Author Year Country	Study type	Specialty or condition/clinician grade or experience	Setting/ recruitment/ Sample size (n)	Uncertainty assessment	Uncertainty Resource	Uncertainty type Cognitive (C) Emotional (E) Ethical (ETH)	Results
Griffiths 2005 UK [13]	Qualitative study	Hormone replacement therapy, bone densitometry and breast screening/Practice nurses, general practitioners, consultants, specialist registrars, specialist nurse, radiographer	7 general practices, 3 secondary care clinics (*n* = 25)	Constant comparative analysis of audio recorded transcripts	Strategies health professionals use	Utilizing safety netting techniques (C) Communicating uncertainty to patients (Eth) Accepting uncertainty (E)	Three key strategies were identified: 1) Focus on certainty for now and this test; 2) providing a coherent account of the medical evidence for the risks and benefits (blurring the uncertainty); and 3) acknowledging inherent uncertainty of medical evidence and negotiating a provisional decision.
Hewson 1996 USA [32]	Process evaluation	Primary and secondary care/a range of clinical experiences (1st year residents to faculty physicians)	Primary and secondary care. 10 tapes of 9 physicians interacting with 4 standardized patient cases in phase one. 19 faculty physicians rating the strategies in phase two.	Clinicians reasoning and strategic medical management was rated using the “Medical checklist, Clinical Reasoning Skills Rating Scale, Interpersonal Skills Rating Scale & Strategic Medical Management Checklist”.	Identification and frequency of strategies used by clinicians when faced with uncertainty	Behaviour patterns when clinicians are faced with diagnostic uncertainty (C) Patient communication and involvement with uncertainty (Eth).	Nine important strategies were identified: 1) defining the context of diagnosis and explaining symptoms; 2) eliminating alternative diagnoses; 3) describing the prognosis; 4) negotiating problems; 5) negotiating the plan of action; 6) keeping diagnostic options open; 7) cautious not to miss potential diagnoses; 8) appropriate time limited safety netting and 9) appropriate contingency planning.
Seaburn 2005 USA [33]	Observational study with 2 unannounced SP visits (thematic analysis)	Family practice / internists and family physicians	Community based primary care in a metropolitan area (*n* = 23); *n* = 46 interviews (the application of 7 codes from thematic analyses led to potentially >46 types of responses).	NA	NA	Greater knowledge about patient’s life circumstances (C) Physician responses to ambiguous symptom presentations by patients (Eth)	Primary care physicians respond to ambiguity by either ignoring the ambiguity and becoming more directive (UC) or, less often, by acknowledging the ambiguity and attempting to explore symptoms and patient concerns in more detail (HP).
Sommers 2007 USA [34]	Intervention evaluation-thematic and frequency analysis	Primary care physicians/NS	Primary care (*n* = 14 practice sites, 98 clinicians with 118 patient cases)	Practice-based learning in small groups	Intervention “Practice Inquiry”	Not knowing enough about the patient and managing clinician-patient boundaries, expectations and trust (C + Eth) Using gut feelings (E)	Of the 30 sites approached between 2002 and 2005, 14 held introductory meetings and by summer 2006, 98 clinicians from 11 sites continued to hold regular Practice Inquiry group meetings suggesting the feasibility and acceptability of the intervention to clinicians.

*SP* Standardized patients, *NA* not applicable, *NS* not stated, *C* Cognitive *E* emotional, *Eth* ethical

All 6 cross-sectional studies were low to moderate quality meeting 3 to 5 of the 7 criteria listed in the modified Newcastle Ottawa scale for cross-sectional studies (please see Additional file 2: Table S1). The qualitative study with high critical appraisal ratings (8 of the 10 criteria) identified issues across all three domains of diagnostic uncertainty which were consistent with the pattern of findings identified by the remaining qualitative and cross-sectional studies. The quality of the qualitative research was generally low to moderate. Three studies (the process evaluations) met 5 criteria while one qualitative study met 8 of the 10 criteria listed in the CASP checklist for qualitative research (please see Additional file 3: Table S2).

The majority of studies [4, 6, 10, 13, 29–33] investigated internal resources or traits/attributes for managing diagnostic uncertainty which included individual traits such as gender, experience, ethnicity, as well as the use of specific strategies used by clinicians such as skills, reasoning styles, rules of thumb and sharing the dilemma with a colleague whilst the only external resource identified related to a specific intervention in the form of an adapted Practice-based learning training program [34].

The level of detail provided in relation to how diagnostic uncertainty was managed ranged from limited preset responses in surveys to in-depth qualitative descriptions. Despite the heterogeneity with regards to their study designs and aims, the studies nonetheless provide some empirical evidence on the types of diagnostic uncertainty currently studied in the literature (emotional, ethical and cognitive) and the impact of specific characteristics on how diagnostic uncertainty manifests and on how diagnostic uncertainty is managed.

### Cognitive aspects of managing uncertainty

Eight studies reported on various cognitive aspects of managing uncertainty [4, 10, 13, 29, 31–34].

Across the qualitative studies, two studies focused on the consideration of the individual patient and their unique biological, psychological and social situations whereby greater knowledge regarding the patient was one strategy by which diagnostic uncertainty could be reduced [30, 31]. Sommers et al. [34] reported how the vast majority of the clinical dilemma cases were categorized as such due to clinician-patient relationships and often involved simply “not knowing” on various fronts. Ensuring knowledge gaps were minimized and having access to a supportive environment with colleagues to reflect and build a trusting network were some of the suggested approaches to deal with cognitive uncertainty [34]. Three studies reported the use of appropriate safety netting techniques particularly using the ‘test of time [13, 32, 34]. Finally, only one study provided a form of training platform to help clinicians deal with diagnostic uncertainty [34]. From 2001 to 2006, Sommers and colleagues developed small and voluntary practice-based learning groups to help clinicians deal and learn from real life case-based clinical uncertainties which included diagnostic uncertainties [34]. From the clinical group discussions of dilemma cases, clinicians were willing to reveal knowledge gaps, cognitive biases and unrealistic expectations, with the former two being associated to diagnostic uncertainties. The study demonstrated the value and usefulness of such voluntary learning groups (particularly being with colleagues) as well as the feasibility and long term sustainability which resulted in 98 clinicians partaking across 11 sites for over a period of 5 years. However, the study did not report any effects of the intervention on the ability of the clinicians at an individual, or group level, to manage their uncertainties more effectively.

The results of four quantitative cross-sectional studies [4, 10, 29, 31] were consistent and complementary to the qualitative data. Evans and Trotter [4] found that the physician’s epistemological stance (biomedical vs psychosocial model) can influence the cognitive thought processes ultimately impacting on how diagnostic uncertainty is managed. Nevalainen demonstrated how as part of their cognitive thought processes, younger GPs found electronic databases more useful than experienced GPs (100% (95% CI 95.8–100.0) vs. 93.7 (95% CI 85.8–97.9), (*p* = 0.018) [29]. In one study, Schneider and colleagues report how intolerance to uncertainty correlates with self-rated diagnostic activity such as the increased ordering of tests [10]. Finally, in another study, Schneider demonstrates how various personality traits from the Big Five inventory (BFI-K) are also associated with diagnostic reasoning (see Table 1) [31].

### Emotional management of uncertainty

Eight of the studies also briefly alluded to different emotional precursors and responses to managing uncertainty [4, 10, 13, 29–32, 34].

Accepting diagnostic uncertainty as an inevitable part of general practice and going with a provisional decision including using intuition and gut feelings was one strategy discussed across several qualitative and cross-sectional studies [10, 13, 31, 32, 34]. Cross-sectional studies also provided additional interesting findings in relation to the emotional management of diagnostic uncertainty. Cooke [30] demonstrated that positive states in physicians such as resilience, is associated with higher levels of compassion satisfaction and personal meaning in patient care. Conversely, low resilience correlated with secondary traumatic stress, inhibitory anxiety, general intolerance to uncertainty and concerns about bad outcomes. In a similar survey administering the “anxiety due to uncertainty” and “concern about bad outcomes” scales; Evans and colleagues [4] found that a biomedical epistemology is associated with more stress reactions to diagnostic uncertainty whilst a psychosocial epistemology is associated with less stress reactions to diagnostic uncertainty. In another survey, Schneider [10] suggests that affective reactions to diagnostic uncertainty may have a consistent emotional and behavioral dimension to it. They also go on to suggest that anxiety may exert gender specific reactions to diagnostic uncertainty in different ways. Female GPs stated higher anxiety due to diagnostic uncertainty (*P* < 0.01) resulting in the use of more primary care heuristics. Male GPs with higher anxiety due to diagnostic uncertainty on the other hand used fewer primary care heuristics and increased test ordering [10]. Nevalainen and colleagues [29] report that experienced GPs tolerate diagnostic uncertainty better than their less experienced counterparts with younger GPs more frequently expressed fears of committing medical errors. They also demonstrate that experienced and less experienced GPs differ with their specific coping strategies. For example experienced GPs were more likely to apologize to the patient about a medical error while their less experienced counterparts were more likely to discuss errors with colleagues instead. Finally, in a more recent survey, Schneider and colleagues [31] demonstrated how individual personality traits from the Big 5 Inventory (neuroticism, extraversion, openness, agreeableness, conscientiousness) were positively associated with diagnostic reasoning techniques as well as communication with patients. For example, physicians scoring high in neuroticism showed more anxiety due to diagnostic uncertainty whereas extraversion, conscientiousness and openness correlated negatively with anxiety due to diagnostic uncertainty [31].

### Ethical management of uncertainty

Eight studies highlighted ethical aspects of diagnostic uncertainty management [6, 13, 29–34].

Across the cross-sectional studies, Portnoy [6] demonstrated that physicians’ perceptions of their patients’ responses to ambiguity influenced their decisions to share that ambiguity. Physicians who thought that more of their patients would have negative reactions to ambiguous information were more likely to decide what’s best for the patient (*p* = 0.013) and to withhold an intervention that had diagnostic uncertainty associated with it (p = <0.001). Nevalainen [29] reported a reluctance to disclose uncertainty and medical errors to patients by less experienced GPs and how experienced GPs were more likely to discuss diagnostic errors with patients [29]. Moreover, in a cross-sectional quantitative survey of Australian registrars [30], showed that intolerance of uncertainty (which included diagnostic uncertainty) and reluctance to disclose uncertainty to patients were associated with a higher degree of burnout [30]. Similarly, Schneider [31] illustrated how GPs scoring high in neuroticism had a higher reluctance to communicate their uncertainties with patients and extraversion, conscientiousness and openness correlated negatively with patient communication [31]. Moreover, Seaburn and colleagues [33] using the quantitative data collected as part of their observational study, identified two distinct responses to diagnostic uncertainty. In a small proportion of GP consultations, GPs avoided acknowledging diagnostic uncertainty or uniformly denied uncertainty (*n* = 13; 22%). Their responses often involved premature and multiple diagnoses, the inability to respond directly to patient questions and concerns, arriving at treatment plans with little or no shared decision-making and occasionally communicating in a way that ignored the patient’s concerns. A greater proportion of GPs acknowledged uncertainty in their consultations (*n* = 48; 77%). They spent more time gathering information, offered support in the form of empathy and completed the examination prior to suggesting potential diagnoses and the diagnostic uncertainties associated with them [33].

The negative impact of failures on disclosing diagnostic uncertainty has also been evident in the qualitative data. Sommers [34] demonstrated how recurring relationship dilemmas between the GP and patient contributed to uncertainties (which included diagnostic uncertainties) amongst their cases. They included negotiating clinician–patient boundaries, aligning patient–clinician expectations and establishing trust with the patient. Finally, Hewson [32] and Griffiths [13] go on to provide a comprehensive account of key skills and strategies (see Table 2) necessary to help manage uncertainty but highlight the need for a more patient-centered approach to managing uncertainty which includes improved communication and attending to patient concerns and fears.

## Discussion

### Summary

This review has provided an overview of the existing empirical literature on managing diagnostic uncertainty in primary medical care. In total, ten studies were identified as meeting the inclusion criteria and indicate that of the existing work in this area, studies have predominantly focused on traits associated with or internal resources of primary medical care clinicians for managing diagnostic uncertainty. Due to the heterogeneity, low quality (which was 1) with respect to the hierarchy of evidence and 2) moderate in terms of their conduct); and paucity of studies in this area, it is unclear however which types of internal resources are most effective for managing diagnostic uncertainty and which domains of uncertainty are most troublesome and/or amenable to intervention. Furthermore, only one external resource/intervention was identified, a training programme, which although acceptable to clinicians reported no evidence on its effectiveness in helping clinicians to better manage their diagnostic uncertainties.

Clinicians in primary care manage and deal with diagnostic uncertainty in a wide variety of ways. As a platform for work in this area, we have categorized the exiting literature on managing diagnostic uncertainty into three domains: cognitive, emotional and ethical domains. By categorizing these elements of diagnostic uncertainty, researchers, medical educators and clinicians may be able to better conceptualize the various components of diagnostic uncertainty enabling them to have an improved awareness of their existence, their potential impact(s) and ultimately to address them. We now briefly discuss some of the issues in each of these domains.

In the ethical domain, there is a need to balance paternalism and truly shared decision-making [12]. Deciding on the level of detail to communicate, finding appropriate descriptors to explain the diagnostic decision and risk – all in the context of infinitely varying degrees of patient expectations and understanding makes the process and unenviable task. This is further exacerbated with few guidelines or tools existing to help clinicians communicate diagnostic uncertainties with their patients. Despite the difficulties, in order to involve the patient in shared decision making, it is essential to share uncertainties with patients regardless of how difficult the clinician may perceive the task to be. A transparent consultation is morally and ethically obligated, is more likely to engage with the patient and lead to better outcomes for the patient and clinician [35]. Indeed, although there are well established tools for assessing communication in health care, none of these are focused primarily on discussions around diagnosis [36].

In the emotional domain, clinicians need to have an increased self-awareness of their own emotional responses to diagnostic uncertainty [37] as well as the potential influence of their individual characteristics and personality traits which may influence how they manage and perceive uncertainty. However, the lack of formal support mechanisms to help clinicians cope with the emotional drain of dealing with diagnostic uncertainties is of particular concern. Indeed, clinicians’ maladaptive responses to uncertainty contribute to work-related stress [38]. More than two decades ago, Young and Spencer [39] argued for formal structured stress and emotional support for GPs and there appears to have been little progress. It would appear that one of the primary avenues for such support in the UK presents in the form of Balint Groups [40], the purpose and aims of which closely resembles the article by Sommers [34].

In the cognitive domain, most research and practice has focused on attempting to aid the cognitive aspects of diagnosis via the development and use of decision aids [41], differential diagnostic (DDX) generator tools [24] or diagnostic guidelines [42]. It is unclear however whether such approaches have succeeded in reducing uncertainty [43]. Furthermore, even the apparent cognitive clarity of dichotomous, quantitative diagnostic tests, may do little to alleviate any diagnostic uncertainty in an environment as messy as primary care where much diagnostic information is lost, as disease prevalence is low and the accuracy of the test is poor [44].

Finally, although we have categorized the included studies into three separate domains of diagnostic uncertainty, as the studies illustrate, the domains are often related and uncertainty is experienced across all or some domains for different individuals and in different scenarios. No studies have attempted to look at this variation.

### Research and policy implications

Clinicians are aware in their everyday practice of the inevitability of diagnostic uncertainties yet the culture of medicine promotes the appearance of certainty, believing it to be for the good of the science, patients and the system. However, as we have discussed, clinicians’ maladaptive responses to diagnostic uncertainty have implications for clinicians themselves, their patients and the health system they work in. Therefore an essential first step must be the acknowledgment and acceptance of the inevitability of diagnostic uncertainties in an area of medicine such as primary medical care [21]. Medical students, trainees and qualified clinicians should be taught to expect diagnostic uncertainty to be part of their work and then ways of effectively managing the various aspects of that uncertainty. Specifically, teaching clinicians to recognize which domains(s) of uncertainty is affecting them at that particular time point and giving them the skills to manage that particular issue/level is paramount. Indeed, we argue that nationally and internationally, all medical curricula be embedded with specific components focusing on managing diagnostic uncertainty and uncertainty more widely, in order to allow prospective doctors to identify which of the domains they are experiencing, when and how to effectively manage those uncertainties. At present it is not particularly a focus for medical education curricula or continuing medical education (CME) [45] and this needs to change. The problem here however, as our review has demonstrated, is that the educational knowledge and training programs required to do this and the good quality research underpinning them, is lacking. Some work has been done on managing uncertainty in medicine more generally [46, 47] but it is not clear if this work is transferable to diagnostic uncertainty, and what if anything is unique about diagnostic uncertainty as opposed to other aspects of uncertainty in medicine. Although changes across CME and medical education curricula could aid both trainee and experienced GPs, it is also unclear to what extent, in which ways, and which groups, if any, would benefit from training in managing diagnostic uncertainty. Some evidence from studies included in this review suggested that physicians with low clinical experience might encounter more difficulties coping with medical errors and diagnostic uncertainty compared to experienced physicians [29]. Future studies aiming to explore the relationship between physician experience and physician outcomes such as burnout would be of clear interest here.

Finally, other possible solutions to managing diagnostic uncertainty exist. For example, the use of DDX tools for cognitive aspects of diagnostic uncertainty, there is no evidence to say whether DDX tools do in fact do this. The research in this area appears to have focused on the diagnostic accuracy of the tools [24, 48, 49] rather than the *impact* of the tools and their use on the diagnostic uncertainty experienced by the clinicians themselves. For example, does the confirmation of the appearance of a clinician’s working diagnosis, in the differential list generated by a DDX tool help to reduce cognitive uncertainty? One could hypothesize that the opposite is true given that the lists can be expansive. Furthermore, even if these tools help clinicians manage diagnostic uncertainty, it is unclear whether this is a more effective approach than training clinicians in different approaches to managing the various types of diagnostic uncertainty. A clear potential initial avenue for research in this area therefore, is to assess the effectiveness of currently available solutions such as DDX tools and to develop methods, tools and/or training programs which aim to teach clinicians to effectively manage diagnostic uncertainty in each of the uncertainty domains. In summary, the scope of research in this area is vast and here we have simply made some initial suggestions of areas for exploration.

### Strengths and limitations

This systematic critical review had several strengths. It constitutes the first attempt to identify and conceptualize the available literature on managing diagnostic uncertainty in general practice as well as that literature into relevant domains which provides a systematic framework for the development of comprehensive interventions directed at managing or reducing diagnostic uncertainty.

We published our study protocol a priori in PROSPERO. Abstract and full paper screening were independently conducted by two authors with a high degree of inter-rater reliability and the study eligibility criteria in terms of study designs were broad and allowed flexibility to include a wide range of studies. Conversely, the significant heterogeneity, the low-moderate critical appraisal ratings and the limited number of studies retrieved, whilst demonstrating the lack of empirical studies on the topic area were a limitation and as such the findings need to be interpreted with this in mind. Moreover, the aims and objectives of the included studies were not always directly addressing our research question but were focused on the broader area of diagnostic uncertainty that met our inclusion criteria. As such there is the potential risk of reporting bias within the primary studies. For example, a survey may not have included and/or reported on all the three levels of interest to us as it was not part of their objectives.

## Conclusions

Primary medical care clinicians are routinely exposed to diagnostic uncertainty. Despite the documented influence on the individual clinicians, their patients and the healthcare systems they work in, this review has demonstrated that the empirical evidence on managing diagnostic uncertainty is extremely limited and of moderate quality. Existing attempts to deal with diagnostic uncertainty have been focused on how to reduce it, rather than training physicians in how to manage or tolerate it. However, if we accept that diagnostic uncertainty is inevitable in primary care, training physicians on how to manage uncertainty at a cognitive, emotional, and ethical domain, will safeguard the quality and cost-effectiveness of the care they will provide. The three levels of diagnostic uncertainty discussed in this review are helpful in providing a platform for the development of such training and further research in this area.

### What is already known on this topic

Clinicians in primary medical care are routinely confronted with diagnostic uncertainties. Intolerance to diagnostic uncertainty is increasingly acknowledged to have negative implications for the primary care practitioner, their patients and the wider healthcare system. However no reviews to date have summarized the existing empirical literature on how diagnostic uncertainty manifests and is managed.

### What this study adds

This is the first review to summarise and conceptualise the existing empirical literature on managing diagnostic uncertainty in primary medical care. Included studies were categorized into three domains (cognitive, emotional and ethical) in order to provide a conceptual basis for the development of future theory based interventions. Finally, these domains are not mutually exclusive, in fact are seemingly often related and all have the potential to impact on the primary care practitioner, their patients and the wider healthcare system if not effectively managed. We propose that these three domain form the basis for future research and what form that research should take, in learning how to manage diagnostic uncertainty in primary medical care.

## Additional files

Additional file 1: Search strategy. The search strategy run in the electronic databases used for the review. (DOCX 16 kb)

Additional file 2: **Table S1.** Quality appraisal of cross-sectional studies. This table demonstrates the quality of the cross-sectional studies included in the review. (DOCX 14 kb)

Additional file 3: **Table S2.** Quality appraisal of qualitative studies. This table demonstrates the quality of the qualitative studies included in the review. (DOCX 14 kb)

## Abbreviations

BFI-K: Big Five inventory
CASP: The Critical Appraisal Skills Programme checklist
CI: Confidence interval
CME: Continuing medical education
DDx: Differential diagnosis
GPs: General Practitioners
MeSH: Medical subject headings
NHS: National Health Service
NPSA: National Patient Safety Agency
PRISMA: Preferred Reporting Items for Systematic Reviews and Meta-Analyses
