# Supplementary material for: Managing diagnostic uncertainty in primary care: a systematic critical review
Source: BMC Fam Pract. 2017 Aug 7;18:79. doi: 10.1186/s12875-017-0650-0 (PMC5545872; doi:10.1186/s12875-017-0650-0)
Supplement: Supplementary file 1 — Search strategy. The search strategy run in the electronic databases used for the review. (DOCX 16 kb) [file 12875_2017_650_MOESM1_ESM.docx]

Search Strategy:

Database: EBM Reviews - Cochrane Database of Systematic Reviews <2005 to April

2015>, EBM Reviews - ACP Journal Club <1991 to May 2015>, EBM Reviews -

Database of Abstracts of Reviews of Effects <2nd Quarter 2015>, EBM Reviews -

Cochrane Central Register of Controlled Trials <April 2015>, EBM Reviews -

Cochrane Methodology Register <3rd Quarter 2012>, EBM Reviews - Health

Technology Assessment <2nd Quarter 2015>, EBM Reviews - NHS Economic

Evaluation Database <2nd Quarter 2015>, AMED (Allied and Complementary

Medicine) <1985 to May 2015>, Embase <1980 to 2015 Week 21>, Global Health

<1973 to 2015 Week 21>, Health and Psychosocial Instruments <1985 to April

2015>, HMIC Health Management Information Consortium <1979 to March 2015>,

Ovid MEDLINE(R) <1946 to May Week 4 2015>, PsycINFO <1806 to May Week 4

2015>

--------------------------------------------------------------------------------

1 Intervention.mp. [mp=ti, ab, tx, kw, ct, ot, sh, hw, tn, dm, mf, dv, bt, id, cc, ac, de,

md, sd, so, nm, kf, px, rx, ui, tc, tm] (1253374)

2 Training.mp. [mp=ti, ab, tx, kw, ct, ot, sh, hw, tn, dm, mf, dv, bt, id, cc, ac, de,

md, sd, so, nm, kf, px, rx, ui, tc, tm] (984186)

3 course*.mp. [mp=ti, ab, tx, kw, ct, ot, sh, hw, tn, dm, mf, dv, bt, id, cc, ac, de, md,

sd, so, nm, kf, px, rx, ui, tc, tm] (1582923)

4 Teaching.mp. [mp=ti, ab, tx, kw, ct, ot, sh, hw, tn, dm, mf, dv, bt, id, cc, ac, de,

md, sd, so, nm, kf, px, rx, ui, tc, tm] (468121)

5 Dealing.mp. [mp=ti, ab, tx, kw, ct, ot, sh, hw, tn, dm, mf, dv, bt, id, cc, ac, de, md,

sd, so, nm, kf, px, rx, ui, tc, tm] (121935)

6 coping.mp. [mp=ti, ab, tx, kw, ct, ot, sh, hw, tn, dm, mf, dv, bt, id, cc, ac, de, md,

sd, so, nm, kf, px, rx, ui, tc, tm] (180213)

7 module*.mp. [mp=ti, ab, tx, kw, ct, ot, sh, hw, tn, dm, mf, dv, bt, id, cc, ac, de,

md, sd, so, nm, kf, px, rx, ui, tc, tm] (103089)

8 brochure.mp. [mp=ti, ab, tx, kw, ct, ot, sh, hw, tn, dm, mf, dv, bt, id, cc, ac, de,

md, sd, so, nm, kf, px, rx, ui, tc, tm] (3478)

9 program.mp. [mp=ti, ab, tx, kw, ct, ot, sh, hw, tn, dm, mf, dv, bt, id, cc, ac, de,

md, sd, so, nm, kf, px, rx, ui, tc, tm] (1290937)

10 Programme.mp. [mp=ti, ab, tx, kw, ct, ot, sh, hw, tn, dm, mf, dv, bt, id, cc, ac,

de, md, sd, so, nm, kf, px, rx, ui, tc, tm] (242833)

11 leaflet*.mp. [mp=ti, ab, tx, kw, ct, ot, sh, hw, tn, dm, mf, dv, bt, id, cc, ac, de,

md, sd, so, nm, kf, px, rx, ui, tc, tm] (40012)

12 management.mp. [mp=ti, ab, tx, kw, ct, ot, sh, hw, tn, dm, mf, dv, bt, id, cc, ac,

de, md, sd, so, nm, kf, px, rx, ui, tc, tm] (2820135)

13 Doctor.mp. [mp=ti, ab, tx, kw, ct, ot, sh, hw, tn, dm, mf, dv, bt, id, cc, ac, de, md,

sd, so, nm, kf, px, rx, ui, tc, tm] (187222)

14 GP.mp. [mp=ti, ab, tx, kw, ct, ot, sh, hw, tn, dm, mf, dv, bt, id, cc, ac, de, md,

sd, so, nm, kf, px, rx, ui, tc, tm] (102765)

15 clinician.mp. [mp=ti, ab, tx, kw, ct, ot, sh, hw, tn, dm, mf, dv, bt, id, cc, ac, de,

md, sd, so, nm, kf, px, rx, ui, tc, tm] (121051)

16 physician.mp. [mp=ti, ab, tx, kw, ct, ot, sh, hw, tn, dm, mf, dv, bt, id, cc, ac, de,

md, sd, so, nm, kf, px, rx, ui, tc, tm] (618756)

17 resident.mp. [mp=ti, ab, tx, kw, ct, ot, sh, hw, tn, dm, mf, dv, bt, id, cc, ac, de,

md, sd, so, nm, kf, px, rx, ui, tc, tm] (125563)

18 intern.mp. [mp=ti, ab, tx, kw, ct, ot, sh, hw, tn, dm, mf, dv, bt, id, cc, ac, de, md,

sd, so, nm, kf, px, rx, ui, tc, tm] (5034)

19 General practitioner.mp. [mp=ti, ot, ab, tx, kw, ct, sh, de, md, sd, hw, tn, dm, mf,

dv, bt, id, cc, ac, ip, vo, pg, jn, yr, ey, cd, cl, pb, sa, ja, bd, ar, bs, cf, dp, dt, pu, lp, pr,

cp, rf, sj, so, mo, op, os, pa, pi, pl, ry, st, nm, kf, px, rx, an, ui, tc, tm] (100960)

20 "uncertaint*".m_titl. (20727)

21 "doubt*".m_titl. (4329)

22 dubiety.m_titl. (4)

23 hesitancy.m_titl. (126)

24 indecision.m_titl. (450)

25 unsure.m_titl. (85)

26 ambiguity.m_titl. (5136)

27 1 or 2 or 3 or 4 or 5 or 6 or 7 or 8 or 9 or 10 or 11 or 12 (7526538)

28 13 or 14 or 15 or 16 or 17 or 18 or 19 (1138745)

29 20 or 21 or 22 or 23 or 24 or 25 or 26 (30707)

30 27 and 28 and 29 (423)

31 remove duplicates from 30 (278)
