# Supplementary material for: Managing diagnostic uncertainty in primary care: a systematic critical review
Source: BMC Fam Pract. 2017 Aug 7;18:79. doi: 10.1186/s12875-017-0650-0 (PMC5545872; doi:10.1186/s12875-017-0650-0)
Supplement: Supplementary file 2 — Table S1. Quality appraisal of cross-sectional studies. This table demonstrates the quality of the cross-sectional studies included in the review. (DOCX 14 kb) [file 12875_2017_650_MOESM2_ESM.docx]

**Additional file 2**

Table S1 Critical appraisal of the quantitative cross-sectional studies using the modified Newcastle Ottawa scale for cross-sectional studies

| Study  Author/Year | Representative sample | Sample size | Ascertain exposure | Non-responders | Comparable outcomes | Assessment of outcome | Follow-up length | Quality ratings |
| --- | --- | --- | --- | --- | --- | --- | --- | --- |
| Cooke 2013 | Yes | No | Yes | No | No | Yes | Yes | Moderate |
| Evans 2009 | No | No | Yes | No | Yes | No | Yes | Low |
| Nevalainen 2014 | No | No | Yes | No | Yes | Yes | Yes | Moderate |
| Portnoy 2011 | Yes | Yes | No | No | Yes | Yes | Yes | Moderate |
| Schneider 2010 | Yes | Yes | No | No | Yes | Yes | Yes | Moderate |
| Schneider 2014 | Yes | Yes | No | No | Yes | No | Yes | Moderate |
