# Supplementary material for: Managing diagnostic uncertainty in primary care: a systematic critical review
Source: BMC Fam Pract. 2017 Aug 7;18:79. doi: 10.1186/s12875-017-0650-0 (PMC5545872; doi:10.1186/s12875-017-0650-0)
Supplement: Supplementary file 3 — Table S2. Quality appraisal of qualitative studies. This table demonstrates the quality of the qualitative studies included in the review. (DOCX 14 kb) [file 12875_2017_650_MOESM3_ESM.docx]

**Additional File 3**

Table S2 Critical appraisal of the qualitative studies using the Critical Appraisal Skills Programme (CASP) checklist for qualitative research

| Study  Author/Year | 1 | 2 | 3 | 4 | 5 | 6 | 7 | 8 | 9 | 10 | Quality ratings |
| --- | --- | --- | --- | --- | --- | --- | --- | --- | --- | --- | --- |
| Grifiths 2005 | Yes | Yes | Yes | Unclear | Yes | No | Yes | Yes | Yes | Yes | High |
| Hewson 1996 | Yes | Yes | Yes | No | No | Yes | Unclear | Unclear | Yes | Yes | Moderate |
| Seaburn 2005 | Yes | Yes | Yes | Unclear | No | Yes | No | No | Yes | Yes | Moderate |
| Sommers 2007 | Yes | Yes | No | No | Yes | No | Unclear | Unclear | Yes | Yes | Moderate |
